# Challenging the Biomimetic Promise 2.0: Negative Spillover of Bio-Inspired Versus Sustainability Framing on Public Perceptions of Bio-Inspired Technologies

**DOI:** 10.3390/biomimetics11030222

**Published:** 2026-03-19

**Authors:** Julius Fenn, Michael Gorki, Stephanie Bugler, Roland Thomaschke, Christian Böffel, Andrea Kiesel

**Affiliations:** 1 Cluster of Excellence *liv*MatS @ FIT–Freiburg Center for Interactive Materials and Bioinspired Technologies, University of Freiburg, Georges-Köhler-Allee 105, 79110 Freiburg, Germany; michael.gorki@livmats.uni-freiburg.de (M.G.); stephaniebugler98@gmail.com (S.B.); kiesel@psychologie.uni-freiburg.de (A.K.); 2 Institute for Psychology, University of Freiburg, 79085 Freiburg im Breisgau, Germany; thomaschke@psychologie.uni-freiburg.de; 3 Work and Engineering Psychology, Institute of Psychology, RWTH Aachen University, 52072 Aachen, Germany; christian.boeffel@psych.rwth-aachen.de

**Keywords:** biomimetic promise, natural-is-better bias, bio-inspired technologies, sustainability, public perception, bio-inspired framing

## Abstract

This study investigates how bio-inspired versus sustainability-focused framing influences lay evaluations of a specific bio-inspired building-technology scenario, testing the empirical validity of the so-called “biomimetic promise”. Employing a between-subjects experimental design (N=582), we examined assessments of a weather-responsive self-shading façade across bio-inspired, sustainable, and neutral framing conditions. We developed and validated the 12-item Perceived Bio-Inspiration Scale (PBS)—a novel standardized psychometric instrument designed to quantify lay recognition of biomimetic features across visual, intentional, and naturalistic dimensions. While results showed robust direct framing effects, we identified a significant negative spillover: emphasizing biological inspiration significantly reduced the technology’s perceived sustainability, while sustainability framing diminished its perceived bio-inspiration. These findings demonstrate, in this façade context, that laypersons evaluate bio-inspiration and sustainability as cognitively distinct and potentially competing constructs, indicating that “natural-is-better” bias is not universal across all technology domains. Consequently, merely invoking biological origins is insufficient to enhance a technology’s ecological appeal. To foster public trust, science communication should shift from abstract biological metaphors toward a performance-driven communication strategy that prioritizes the disclosure of verifiable life-cycle assessment and specific operational advantages over symbolic nature-based analogies.

## 1. Introduction

Tackling the global challenges of climate change and sustainability remains one of the most urgent tasks of the Anthropocene [1]. In this context, bio-inspired technologies (despite the article explains the concept of biomimicry, it is important to note that there are several related yet distinct bio-inspired design approaches, including bionics, biomimetics, and biomimicry, which are summarized under the broader term Biom* [2]. Bionics derives technical principles from biological systems without necessarily imitating them; biomimetics imitates biological structures or functions but does not intrinsically incorporate sustainability; and biomimicry uniquely positions nature as model, measure, and mentor, explicitly embedding ecological sustainability within its design methodology [2,3]) have gained prominence as potential low-impact, efficient, and innovative alternatives to conventional engineering approaches [4,5,6]. More broadly, bio-inspired design spans a spectrum of approaches that differ in both epistemic aim and normative scope: in engineering-oriented biologically inspired design, the central activity is often the abstraction of functional principles from biology and their translation into human devices and processes, primarily to improve technical performance or enable new functionalities [7,8,9]. By contrast, in some sustainability-oriented biomimicry discourses, biological systems are not only treated as sources of functional inspiration but also as reference points for evaluating the ecological appropriateness of technology [2,10,11]. Biomimicry as a design methodology and conceptual approach derives and translates functional principles from biological systems into engineered technologies by emulating their structures, mechanisms, or processes, with the aim of improving technical performance while (at best) enabling more resource-efficient and potentially more sustainable solutions [2,3,10]. Such bio-inspired technologies draw on principles distilled from biological systems—such as iterative experimentation, continuous operation during adaptation, and the preservation of useful past solutions—they offer design strategies shaped by millions of years of evolutionary refinement [12]. Thereby, biomimicry positions nature as a model (studied to derive design principles for solving human problems), a measure (used as an ecological standard for assessing what works, what is appropriate, and what lasts), and as a mentor (serving as a source of learning rather than extraction) [10]. However, this formulation reflects a particular sustainability-oriented interpretation of biomimicry rather than a consensus across biomimetics and bio-inspired design more broadly [2,13]. In many engineering contexts, including biomimetics and bio-inspired robotics, biological systems are used primarily as epistemic and functional resources for improving performance, while environmental benefits remain an empirical question to be demonstrated through performance evaluation, life-cycle assessment, or systems-level analysis rather than inferred from biological origin alone [3,5,13].

This triad of Benyus [10] has been interpreted in diverse ways across biomimicry and biomimetics discourses [13,14]: in many accounts, nature functions primarily as a technical model that provides functional principles to be abstracted and translated into technology, e.g., to improve performance or enable new functions [3]. In other accounts—especially those aligned with sustainability-oriented biomimicry discourses—nature is additionally framed as a normative source of ecological principles for evaluating the ecological appropriateness of human technologies [2,11,15,16]. In this version of biomimicry, nature is invoked not only as an epistemic resource (“how to design”), but also as an ecological benchmark for “designing appropriately within the biosphere”, often summarized in the formulation of nature as “model, measure, and mentor” [10]. However, this normative interpretation is contested and does not reflect a field-wide consensus: across biomimetics research, sustainability is typically treated as context-dependent and to be demonstrated via performance evaluation, life-cycle or systems-level assessment rather than inferred from biological origin alone [3,5,12]. Against this backdrop, we use the term *biomimetic promise* to denote a sustainability-oriented discourse expectation—prominent in some practitioner and public narratives—that learning from nature will tend to yield ecologically sound, low-risk, and resource-efficient innovations [4,17].

Our study does not assume that this expectation is technically valid, as the biomimetic promise is not automatically fulfilled: (i) sustainability is a human-defined goal and evolution is non-teleological [3,12], (ii) biological systems are not inherently optimized or sustainable, as evolutionary processes yield context-dependent trade-offs and suboptimal solutions that may be undesirable when transferred to technology [18,19], and (iii) biomimetic applications can fail when detrimental biological traits are reproduced, when resource-intensive manufacturing negates ecological benefits, or when biological–technical context mismatches arise [18,20]; rather, we test whether such a promise is mirrored in lay inference as a “natural-is-better” bias [21]. For example, naturalness-bias research shows that people may prefer a “natural” over a synthetic drug even when both are described as equally safe and effective [21]. Importantly, assuming inherent ecological superiority would risk committing the naturalistic fallacy, which erroneously derives a normative “ought” (that a technology is sustainable) from a factual “is” (that it is based on biological systems), thereby conflating a natural description with a value judgment [22]. Nevertheless, in our perspective, the biomimetic promise serves as a productive driver of sustainable innovation [4,5,16]. From a technical perspective, realizing this potential requires systematic validation through sustainability frameworks and Life Cycle Assessments to quantify the actual ecological impact of the biological-to-technical transfer [23,24,25]. A concrete illustration is provided by the lotus-inspired façade paint Lotusan^®^: its environmental advantage was established not by biological inspiration alone, but by comparative life-cycle and product sustainability assessment against the conventional paint Jumbosil^®^, which attributed its better overall performance mainly to longer service life and thus fewer repainting cycles, lower material demand, and lower labor input over the use phase [24].

In the following sections, we explain and experimentally test the empirical validity of this bias within the context of bio-inspired self-shading façade, whereby the present work examines a communication and perception question: how laypersons infer sustainability from bio-inspired versus sustainability-focused framing. We do not assess the technical sustainability of biomimetic designs, nor do we assume consensus among biomimetics researchers about sustainability being intrinsic to bio-inspiration. Instead, we test whether laypeople apply a natural-is-better bias when evaluating a concrete bio-inspired façade scenario.

### The “Naturalness Bias”: Inferring Sustainability from Bio-Inspired Cues

In this article, we therefore use the term biomimetic promise in the sustainability-oriented claim—advanced by some influential actors [10,26]—that learning from nature will yield more sustainable solutions in the sense of an expectation of inherent sustainability. A previous experimental study on laypeople’s perception of biomimetic buildings reports that merely labeling buildings as biologically inspired does not increase laypersons’ perceived sustainability or acceptability, underscoring that public judgments depend on case-specific information and explicit sustainability communication [27]. Therefore, if the biomimetic promise resembles a cognitive bias among laypersons—a tendency to automatically infer sustainability from biological inspiration regardless of actual performance—remains still an open empirical question to be further investigated [27].

In general, evaluating complex or unfamiliar technologies often relies on cognitive heuristics—efficient mental shortcuts that simplify decision-making under uncertainty [28,29]. Rather than conducting exhaustive technical assessments, laypersons possibly utilize salient proximal cues (e.g., visual resemblance to nature, linguistic framing) to infer distal attributes such as sustainability, safety, or efficiency [30]. Brunswik’s Lens Model [31] provides a framework for this inferential process. It distinguishes between the ecological validity of a cue (its objective correlation with a distal trait) and cue utilization (the subjective weight an observer assigns to it) [30].

In the context of the *biomimetic promise*, bio-inspired framing could function as a powerful cue. Invoking natural resemblance or evolutionary optimization may trigger a “naturalness heuristic”, where the attribute of being “bio-inspired” is weighted heavily as a proxy for sustainability, regardless of the technology’s actual life-cycle performance. However, the strength of this cue is context-dependent. While the “natural-is-better” bias is well-documented in products applied to the body, such as food or cosmetics [21,32], its application to more complex technical systems remains unclear. In these domains, bio-inspiration is often a conceptual or functional transfer rather than a material one [3].

Initial evidence suggests that the biomimetic promise may not be a universal cognitive default; for instance, Gorki et al. [27] found that informing participants of a building’s bio-inspired origins failed to increase its perceived sustainability or acceptance. This discrepancy highlights a significant research gap: it remains unclear whether bio-inspired framing reinforces sustainability perceptions or whether it induces a “negative spillover” effect. In the latter scenario, highlighting bio-inspiration might distract from or even undermine sustainability information by shifting the observer’s evaluative focus. Furthermore, empirical progress is currently limited by the lack of validated instruments to quantify how laypersons perceive the “bio-inspiredness” of a technology itself (see Section 2.4.2).

The present research addresses these gaps by employing two distinct framing conditions—*Bioinspiration* and *Sustainability*—to evaluate their impact on public perception. First, we establish the efficacy of the manipulation by testing whether each frame successfully increases the perceived salience of its targeted dimension (i.e., whether bio-inspired framing elevates bio-inspiration ratings and sustainability framing elevates sustainability ratings). Importantly, we investigate the cross-interaction effects between these constructs. Based on the biomimetic promise, we expected a positive spillover where a bio-inspired framing automatically bolsters sustainability assessments. Conversely, we examine whether a sustainability framing exerts any reciprocal influence on perceived bio-inspiration—an effect that is theoretically less expected. Our primary objectives are:

1. To develop and validate the Perceived Bio-Inspiration Scale (PBS) as a standardized psychometric instrument for measuring lay recognition of biomimetic design features.
2. To experimentally test the directional effects of bio-inspired versus sustainability framing on both target and non-target evaluative dimensions

By employing a controlled vignette design of a bio-inspired façade system, we examine whether these framings facilitate an overall positive bias as predicted by the biomimetic promise or induce a negative spillover that suppresses the very ecological promise they intend to communicate.

## 2. Methods

### 2.1. Design Overview

**Figure 1:**
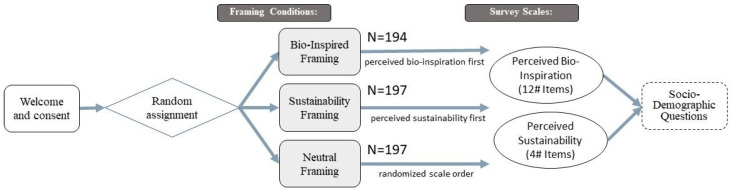
Flow diagram of the experimental design. Participants provided informed consent before being randomly assigned to one of three framing conditions (bio-inspired, sustainability, or neutral). Each framing condition was paired with a specific scale-order arrangement: the bio-inspired framing condition received the Perceived Bio-Inspiration Scale first, the sustainability framing condition received the Perceived Sustainability Scale first, and the neutral condition received a randomized scale order. All participants then completed both outcome measures followed by socio-demographic questions.

We employed a between-subjects experimental design to investigate how framing influences public perceptions of a bio-inspired technology, following a structured five-step procedure illustrated in Figure 1. (1) Participants first provided informed consent. (2) They were then randomly assigned to one of three framing conditions: a bio-inspired framing, a sustainability framing, or a neutral framing. (3) Participants read a vignette describing a weather-responsive, bio-inspired self-shading façade based on pine-cone biomechanics. The factual content was held constant across conditions, with only the framing varied. (4) Following the vignette, participants completed two outcome measures: our newly developed 12-item Perceived Bio-Inspiration Scale (PBS) and the 4-item Perceived Ecological Sustainability Scale (PES). To minimize order effects and potential priming between scales, presentation was condition-specific: participants in the bio-inspired and sustainability groups received the scale corresponding to their specific framing first. Crucially, the neutral condition received a randomized scale order, which served as an internal control to verify that scale sequence did not significantly bias ratings. A preliminary analysis confirmed no significant order effects within the neutral condition (p>0.05), supporting the validity of our presentation strategy. (5) Finally, participants completed socio-demographic questions.

### 2.2. Hypotheses

The present study investigates how bio-inspired versus sustainability framing shapes a layperson’s perception of a biomimetic self-shading façade. Based on the theoretical rationale and the inclusion of a baseline control, we derived the following hypotheses:

**H1a:**  *Exposure to a bio-inspired framing will increase perceived bio-inspiration relative to both sustainability-framed and neutrally framed (control condition) descriptions (see Section 3.3).*

**H1b:**  *Exposure to a sustainability framing will increase perceived sustainability relative to both bio-inspired and neutrally framed descriptions (see Section 3.3).*

**H2:**  *We investigate the potential for cross-dimensional spillover effects between framing and perception. Specifically, we test whether a bio-inspired framing impacts sustainability ratings—an effect expected under the “biomimetic promise”—and conversely, we analyze exploratory whether a sustainability framing influences perceived bio-inspiration as prior research provides no clear predictions regarding the size or direction of such spillovers (see Section 3.4).*

### 2.3. Vignettes

Motivation of methodology. In this study, a *vignette* (i.e., a brief scenario) is operationalized as a short, standardized narrative description of a technology that functions as the experimental stimulus [33]. Vignette-based scenario methods resemble “a set of stories” that help structure perceptions of an (often unfamiliar) technology [34]. Importantly, such scenarios are hypothetical constructs that do not claim to represent reality in full detail; rather, they highlight selected key features to focus attention on the variables of interest [35].

Three standardized vignettes were created to manipulate how the fictional self-shading façade was framed—either as *bio-inspired*, *sustainable*, or *neutral*. All three versions were matched in length, readability, and factual accuracy, and differed exclusively in the framing cues they emphasized. To ensure consistency and scientific rigor, the vignettes were developed through a multi-step procedure involving literature review, AI driven literature search and scenario generation, human editing, and expert validation. The drafting process followed the steps following six steps:

(i) Conducted a literature search of the self-shading façade, synthesizing information from scientific publications, grey literature, and requested experts from the Cluster of Excellence “Living, Adaptive and Energy-autonomous Materials Systems” (*liv*MatS) at the University of Freiburg (Germany) for relevant sources [36].
(ii) Extracted key functional, biological, and sustainability-related attributes to serve as input for vignette generation.
(iii) Generated initial vignette drafts using a Retrieval-Augmented Generation (RAG) pipeline together with a Large Language Model (GPT-4.0), producing initial text of the three framing conditions [37,38]. Methodologically, the purpose of this step was to enforce strict structural equivalence (identical sentence count, parallel syntax, and constant factual core) so that framing was the only manipulated variable. Concretely, we employed a prompt template that (a) specifies a textual structure, (b) constrains the model to use only author-provided factual content (based on RAG and manually derived attributes from previous step), and (c) instructs that evaluative language may appear only where the framing requires it (bio-inspired vs. sustainability). The exact prompt template, version history, and intermediate drafts are provided in the online repository (Materials folder). To address common risks of AI-assisted drafting (e.g., factual inaccuracies, non-deterministic outputs), we implemented three downstream quality-control steps. First, we performed a systematic verification of each vignette against the source-derived attribute list from step (ii) to ensure that all factual statements were accurate. Second, two rounds of manual editing were used to verify content, harmonize tone and readability across conditions, step (iv), (v):
(iv) Performed a preliminary review and manual edits to ensure clarity, accuracy, and consistent tone.
(v) Submitted the vignettes for expert review by researchers at *liv*MatS and RWTH Aachen (author C.B.) and revised and refined the vignettes based on expert feedback, addressing inaccuracies and adjusting text length where needed.
(vi) Finalized the vignettes for use in the experiment after a second round of expert-informed revisions.

Self-shading façades are part of a growing class of biomimetic and bio-inspired strategies that translate adaptive principles from plants and other organisms into responsive architectural systems [19,39]. Examples include dynamic mechanisms such as hinge-less deformation inspired by *Strelitzia reginae* (Flectofin^TM^) and folding movements derived from *Mimosa pudica*, as well as material-driven approaches such as hygromorphic bilayers based on pine cone actuation [40]. Collectively, these systems reflect a broader shift toward adaptive, multifunctional façades designed to autonomously modulate environmental loads and reduce operational energy demand.

**Figure 2:**
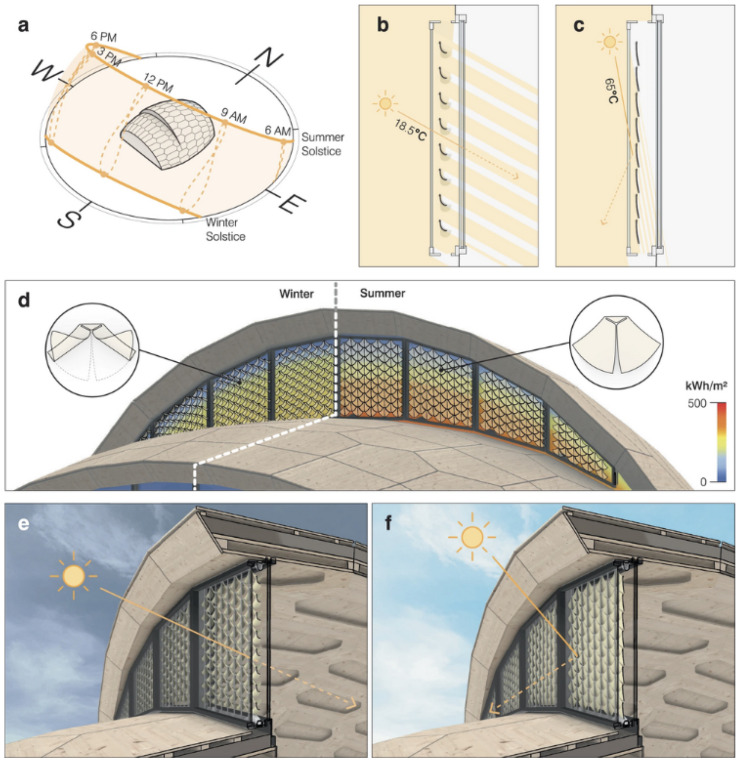
Illustration of a hygromorphic responsive self-shading façade composed of curved modules that autonomously open under cool, humid conditions to admit solar heat and close under warm, dry conditions to provide shade. The system exemplifies passive, material-based actuation as demonstrated in recent cellulose-based façade research. (Image source: © ICD/ITKE/IntCDC Universität Stuttgart, Rob Faulkner from [41]).

The self-shading façade described in the vignettes represents one such adaptive envelope, capable of regulating solar gains through material-based, autonomous actuation. As illustrated in Figure 2, the façade consists of numerous small, curved modules that curl or flatten in response to ambient humidity and temperature, thereby adjusting the entry of light and heat without motors, electronics, or external energy input. This functionality aligns with hygromorphic principles demonstrated in recent work on cellulose-based, 4D-printed façade elements, which exhibit robust seasonal responsiveness, durability, and effective modulation of solar radiation under real-world conditions [41,42].

We chose this self-shading façade as the stimulus because it represents a realistic and documented bio-inspired architectural case, is likely unfamiliar to most lay participants (supporting a meaningful test of cue-based inference under uncertainty), and allows a coherent functional core to be held constant while varying only framing cues. The three vignette conditions differed solely in how this identical functional system was framed. The *neutral framing* provided a purely technical description, focusing on material composition and humidity-responsive movement. The *bio-inspired framing* emphasized the system’s conceptual roots in plant motion—particularly pine cone hygromorphy—meaning that its opening and closing behavior mimics how pine cones naturally open when dry and close when humid [41]. In contrast, the *sustainability framing* highlighted the system’s potential contributions to energy efficiency, resource-conscious material use, and climate-responsive building design (see vignette texts in Appendix A).

### 2.4. Scales

Psychometric rationale and terminology. Because our outcomes (e.g., perceived sustainability and perceived bio-inspiration) are latent constructs that cannot be observed directly, we assess them with multi-item psychometric scales, which reduce measurement error and allow estimation of the underlying construct more reliably than any single item. Exploratory factor analysis (EFA) is applied to examine how many factors are can be discovered by the item co-variance pattern (structure discovery), and confirmatory factor analysis (CFA) to test an explicitly specified measurement model by evaluating how well the model-implied covariance matrix reproduces the observed covariance matrix (structure testing) [43]. CFA model fit is summarized via standard indices that capture complementary aspects of misfit: the Comparative Fit Index (CFI) and Tucker–Lewis Index (TLI) values around CFI/TLI≥0.95, the Root Mean Square Error of Approximation (RMSEA) value of RMSEA≤0.06 and Standardized Root Mean Square Residual (SRMR) value of SRMR≤0.08 are often interpreted as good fit [44,45].

#### 2.4.1. Perceived Ecological Sustainability Scale (PES)

Perceived Sustainability was assessed using the four-item ecological dimension of a sustainability scale, adapted from the environmental value subscale of [46] and successfully applied in a previous study [47]. In the present study, we retain the term perceived ecological sustainability to indicate explicitly that the PES captures only the ecological dimension of sustainability, rather than its social or economic dimensions [48]; it does not denote a full engineering-based sustainability or life-cycle assessment.

The ecological subscale was selected because existing multidimensional sustainability instruments, which assess ecological, social, and economic dimensions, are not readily applicable to brief vignette-based evaluations of specific technologies and often presuppose detailed domain knowledge. For example, comprehensive scales such as those that incorporate social criteria (e.g., fair labor standards or human-rights compliance; cf. [49,50]) require information that cannot be inferred from our short vignettes. The PES captures generalizable, context-independent judgments of environmental performance (see Table 1), enabling respondents to evaluate sustainability even when only limited product information is available. Participants responded on a 7-point Likert scale (1 = strongly disagree, 7 = strongly agree).

Structural Validity. Internal consistency of the four-item ecological scale was high (Cronbach’s α=0.86; McDonald’s ω=0.88), indicating strong homogeneity among items (technical remark: McDonald’s ω was computed using the psych package [51] with a one-factor model and provides a reliability estimate that does not assume tau-equivalence [52,53]). To assess dimensionality, an exploratory factor analysis (EFA) was conducted. Parallel analysis identified a single underlying factor. Accordingly, a one-factor solution emerged with loadings ranging from 0.61 to 0.90 and explaining 68.6% of the variance, providing clear evidence that the items tap a common underlying construct. This structure was subsequently evaluated using confirmatory factor analysis (CFA) (technical remark: The confirmatory factor analysis was estimated as a single-factor model using robust maximum likelihood (MLR) with the lavaan package [54], which yields Huber–White robust standard errors and a Yuan–Bentler scaled $\chi^{2}$ test statistic appropriate for moderately non-normal data [55]. The latent factor variance was fixed to 1.0 for identification, all residuals were initially specified as uncorrelated, and model evaluation relied on robust fit indices). The hypothesized one-factor model showed excellent fit to the data, CFI=1.00, TLI=1.00, RMSEA=0.00 (90% confidence interval: [0.000–0.095]), and SRMR=0.009. Standardized factor loadings ranged from 0.55 to 0.88, all statistically significant (p<0.001), and modification indices did not suggest any theoretically meaningful cross-loadings or correlated residuals. Taken together, according to validation guidelines [43,56], the EFA and CFA results provide evidence for the structural validity of the ecological scale.

#### 2.4.2. Perceived Bio-Inspiration Scale (PBS)

Justification. The PBS was developed because no existing instrument quantifies how laypersons judge the extent to which a specific technology is bio-inspired. Available measures like the Perceived Biophilic Design Scale investigate, across seven distinct factors, how humans comprehend biophilic features and experience human–nature relations within the built environment [57]. Biophilia, in turn, denotes the inherent human affinity for nature and nature-like environments, including emotional, symbolic, and restorative responses to natural settings or biophilic design features [58,59]. Likewise, expert-oriented frameworks for bio-inspired design and biomimetics such as BioTRIZ, which analyzes how biological systems resolve functional contradictions (i.e., opposing performance requirements) to inform engineering solutions [9], the Structure–Behavior–Function models, which formally represent components, causal processes, and functions to support mechanism-based abstraction, and the 6W framework, which hierarchically organizes biological knowledge along the interrogatives what, where, who, how, when, and why to aid bio-inspired knowledge extraction [60], focus on abstracting and transferring biological principles into engineering solutions, but they do not assess whether non-experts actually recognize a design as nature-inspired.

Item Development. The constructed PBS consists of three theoretically grounded factors (see Table 2): Visual Resemblance to Nature (VRtN), Intentionality and Perceived Inspiration (IPI), and Perceived Naturalness (PN). Building on the conceptual foundations outlined above, the item pool was mainly motivated both from biophilia-based accounts of how individuals perceive nature-like features [57,58] and from bio-inspired design frameworks that formalize how biological knowledge is structured and transferred [60]. Each factor was further informed by distinct strands of theory and empirical research: VRtN captures the extent to which a technology is perceived as visually echoing organisms or natural patterns, drawing on biophilia and biophilic design research demonstrating the salience of naturalistic forms and environmental cues in shaping nature-related perception [58,61]. IPI reflects perceived deliberate biological modeling, grounded in theoretical distinctions between inspiration, imitation, and integration as progressively deeper forms of engaging with biological models [16], and empirically supported by evidence that lay observers tend to infer biological intent only when it is explicitly communicated, rather than from visual features alone [27]. PN assesses global impressions of naturalness, motivated by work on the natural-is-better bias, and supported by evidence that perceived naturalness systematically shapes risk evaluation, or aesthetic preference independent of functional understanding [21]. Item formulation followed recommended procedures for scale development [56,62,63], including broad coverage of each construct, the inclusion of reverse-coded items, and removal of overly abstract or technically framed content. Finally, domain experts in biomimetics and psychology reviewed and refined the items to optimize content validity, clarity, and representativeness, leading to iterative improvements such as eliminating a previously included fourth factor (“functional analogy”), which was removed due to concerns regarding content validity. While functional analogies are central to biomimetics, we noted that assessing whether a layperson recognizes such analogies requires technical domain knowledge that cannot be reliably inferred from brief vignettes, thereby risking invalid measurements. Content validity thereby refers to the extent to which an item set adequately represents the full conceptual domain of the construct [64]. On this basis, a final set of 12 items was selected, comprising four items per dimension (one reverse-coded item per dimension to mitigate acquiescence bias).

Structural Validity. The initial four-item model of PN showed poor psychometric fit—including low internal consistency (α=0.54), and a CFA indicating inadequate fit (e.g., RMSEA=0.13) the reverse-coded item (PN2r) was removed prior to re-estimating the scale. Subsequently across all three PBS subscales, internal consistency was satisfactory to high: for VRtN, Cronbach’s α=0.86 and McDonald’s ω=0.86; for IPI, α=0.85 and ω=0.87; and for PN α=0.79 and ω=0.79. Parallel analysis consistently supported a unidimensional structure for each subscale. Corresponding single-factor CFAs estimated with robust maximum likelihood for each dimension corroborated these results, with all models exhibiting excellent global fit (fit indices indicative of essentially perfect fit and standardized factor loadings between 0.45 and 0.89, all p<0.001), and no theoretically meaningful correlated residuals suggested by modification indices. An additional overall EFA across all PBS items yielded a two-factor rather than a three-factor solution, with the two extracted factors showing a substantial inter-factor correlation (r=0.71), suggesting that respondents tend to evaluate bio-inspired technologies in a globally integrated manner—such that higher perceived visual resemblance to nature is typically accompanied by higher perceived biological intentionality and naturalness. Nonetheless, each subscale demonstrated clear internal coherence and distinctiveness in its item-level structure, providing evidence for the structural validity of the three-factor PBS.

## 3. Results

Analytic strategy and effect-size interpretation. To test whether the three framing conditions produced systematic differences in mean ratings, the analyses primarily relied on one-way ANOVAs with Tukey-adjusted post hoc comparisons. Omnibus *F*-tests (testing whether any group means differ) are reported together with an ANOVA effect size ($\eta_{G}^{2}$), which quantifies the proportion of variance in the outcome attributable to the framing manipulation and thereby conveys practical relevance on a scale-independent metric [65,66]. To evaluate the spillover hypothesis, an ordinary least squares (OLS) linear model with a framing × rating-type interaction was estimated. Because outcomes were *z*-standardized prior to model estimation, the regression coefficients are interpretable as standardized mean differences (in SD units), enabling direct comparison of effect magnitudes across PBS and PES despite their different original response scales [65]. For ease of interpretation, standardized mean differences are described using conventional reference ranges (approximately 0.2 = small, 0.5 = medium, 0.8 = large) [67].

### 3.1. Sample Characteristics

A total of 594 participants were recruited from the panel provider Prolific. After the removal of participants who accidentally participated in more than one condition the final sample consisted of 582 participants (45.78 years on average, SD=13.46 and 45.1% female, 54.9% male). The vast majority of participants reported no prior experience with comparable technologies (94.4%). Participants were randomly assigned to one of the three framing conditions with comparable group sizes (bio-inspired n=192, neutral n=193, sustainable n=197). Across conditions, age did not differ significantly as shown by a one-way ANOVA, F(2,585)=0.58, p=0.561, and gender distributions did not vary across framing groups, as indicated by a chi-square test, $\chi^{2}\left(2,N=588\right)=1.17$, p=0.556. Prior experience also showed no significant group differences, $\chi^{2}\left(6,N=588\right)=10.55$, p=0.103, confirming similar demographic characteristics across conditions. Participants spent on average 5.49 min completing the study (SD=3.52) and were compensated at a rate of 13 GBP (approximately 17 USD) per hour.

### 3.2. Descriptive Statistics

Descriptive statistics showed clear and consistent differences across framing conditions for both dependent variables (see Table 3). For the PBS, the bio-inspired framing yielded the highest ratings (M=3.92, SD=0.51), followed by the neutral framing (M=3.48, SD=0.70), and the sustainability framing (M=3.18, SD=0.82). For the PES, the sustainability framing produced the highest ratings (M=5.88, SD=0.84), followed by the neutral framing (M=5.46, SD=0.95), and the bio-inspired framing (M=4.83, SD=1.06).

These patterns align with the framing-specific predictions: bio-inspired framing produced the highest bio-inspiration ratings and the lowest sustainability ratings, whereas sustainability framing produced the highest sustainability ratings and the lowest bio-inspiration ratings. The inferential tests are presented in the next Section 3.3.

### 3.3. Framing Effects

Bio-inspired framing significantly increased perceived bio-inspiration: The one-way ANOVA revealed a robust main effect of framing condition on overall PBS scores, F(2,585)=55.95, p<0.001, $\eta_{G}^{2}=0.161$. Tukey-adjusted post hoc tests showed that the bio-inspired frame elicited significantly higher perceived bio-inspiration than the neutral (ΔM=0.441, SE=0.070, p<0.001) and sustainability frames (ΔM=0.733, SE=0.070, p<0.001), with the neutral frame also yielding higher PBS scores than the sustainability frame (ΔM=0.292, SE=0.070, p=0.0001). These findings indicate that the framing effectively increased perceived bio-inspiration, supporting H1a (see Section 2.2).

Similarly, sustainability framing selectively heightened perceived ecological sustainability: The ANOVA showed a strong main effect of framing condition on PES ratings, F(2,585)=60.78, p<0.001, $\eta_{G}^{2}=0.172$. Tukey post hoc comparisons confirmed that the sustainability frame produced significantly higher perceived sustainability than both the neutral (ΔM=0.419, SE=0.096, p<0.001) and bio-inspired frames (ΔM=1.056, SE=0.096, p<0.001). Additionally, the neutral frame yielded higher PES scores than the bio-inspired frame (ΔM=0.637, SE=0.096, p<0.001). Together, these results support H1b.

The next Section 3.4 examines whether these targeted shifts produce cross-dimensional negative spillover effects, that is, whether emphasizing one dimension (framing) simultaneously reduces judgments on the non-target dimension.

### 3.4. Negative Spillover

**Figure 3:**
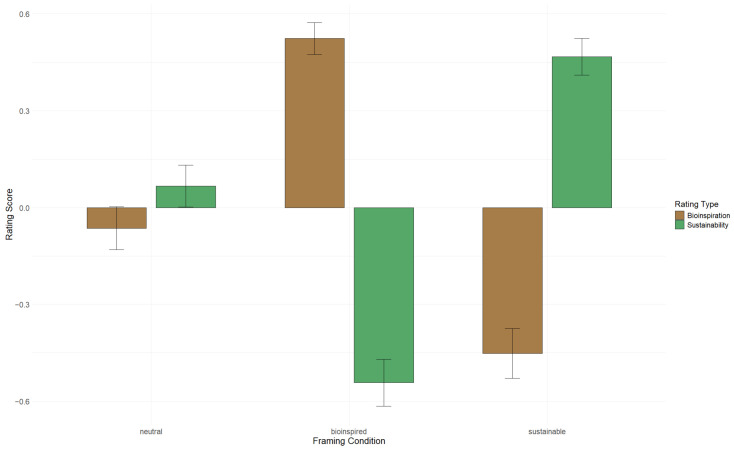
Standardized mean ratings (with standard errors) for perceived bio-inspiration (PBS) and perceived ecological sustainability (PES) across the three framing conditions (neutral, bio-inspired, sustainable). Scores represent *z*-standardized scale means, enabling direct comparison of effects sizes across the scales (Rating Type) and three framing conditions.

To examine whether framing induced cross-dimensional spillover between perceived bio-inspiration (PBS) and perceived ecological sustainability (PES), we first *z*-standardized each scale’s overall mean score across the full sample. Standardization ensured that all effects from the linear model could be interpreted on a common metric (standard deviation units), despite the two scales differing in their original response ranges (1–5 for PBS; 1–7 for PES). Because the outcome variable was standardized, all fixed-effect estimates from the linear model correspond to standardized mean differences (i.e., Cohen’s *d*). Figure 3 shows a clear divergence across the three framing conditions: bio-inspired framing raised perceived bio-inspiration and lowered perceived ecological sustainability, sustainability framing reversed this pattern, and neutral framing produced even ratings.

The data were then reshaped into long format, with *rating type* (PBS vs. PES) treated as a within-participant factor and *framing condition* (neutral, bio-inspired, sustainable) as a between-participants factor. We estimated an ordinary least squares regression (linear model. See Table 4) of the form:

$$\begin{array}{cc} {\mathrm{ratingScore}}_{ij} & =\beta_{0}+\beta_{1}\;{\mathrm{BioFraming}}_{i}+\beta_{2}\;{\mathrm{SustFraming}}_{i}+\beta_{3}\;{\mathrm{PES}}_{j}\\ & \;+\beta_{4}\;\left({\mathrm{BioFraming}}_{i}\times {\mathrm{PES}}_{j}\right)+\beta_{5}\;\left({\mathrm{SustFraming}}_{i}\times {\mathrm{PES}}_{j}\right)+\varepsilon_{ij}, \end{array}$$

with neutral framing (between-participant factor) and PBS (within-participant factor) serving as reference levels. The model explained a modest proportion of variance in the standardized ratings, F(5,1170)=46.68, p<0.001, adjusted $R^{2}=0.163$, providing an appropriate basis for evaluating whether framing influenced PBS and PES ratings to different degrees.

In detail the analysis revealed strong and directionally consistent main effects of framing on PBS: bio-inspired framing elevated PBS scores by 0.587 SD, p<0.001, whereas sustainable framing depressed PBS scores by 0.388 SD, p<0.001, relative to neutral framing. Importantly, under neutral framing, PBS and PES were statistically indistinguishable (Δ=0.131 SD, p=0.157), indicating that participants did not inherently differentiate between the two constructs in the absence of a framing.

The critical evidence for spillover appeared in the interaction terms. The bio-inspired × PES interaction was strongly negative, β=−1.197 SD, p<0.001, indicating that bio-inspired framing substantially reduced PES ratings relative to PBS ratings. Conversely, the sustainable × PES interaction was positive, β=0.789 SD, t(1170)=6.05, p<0.001, reflecting that sustainable framing substantially increased PES ratings relative to PBS ratings. Together, these interactions represent a pronounced crossover pattern.

Estimated marginal means (EMMs) illustrate this divergence clearly. Under bio-inspired framing, participants rated the target as markedly bio-inspired (z=0.524) but markedly unsustainable (z=−0.543). Under sustainable framing, the relationship reversed: sustainability ratings were elevated (z=0.467) while bio-inspiration ratings were depressed (z=−0.452). Neutral framing produced means close to zero (PBS: z=−0.064; PES: z=0.067). The difference between rating types within each framing condition was large: within bio-inspired framing, PBS exceeded PES by 1.066 SD, p<0.001; within sustainable framing, PES exceeded PBS by 0.919 SD, p<0.001. In contrast, the difference under neutral framing was not significant.

Taken together, these results provide convergent evidence for *negative spillover*: bio-inspired framing decreased perceptions of ecological sustainability, and sustainability framing decreased perceptions of bio-inspiration. All effects were large (approaching or exceeding 1 SD in many cases), and statistically significant. Contrary to the biomimetic promise—which would predict synergistic uplift across PBS and PES (at least in the bio-inspired framing condition)—participants instead evaluated bio-inspiration and sustainability as separable, and at times contradictory, evaluative dimensions.

## 4. Discussion

**Figure 4:**
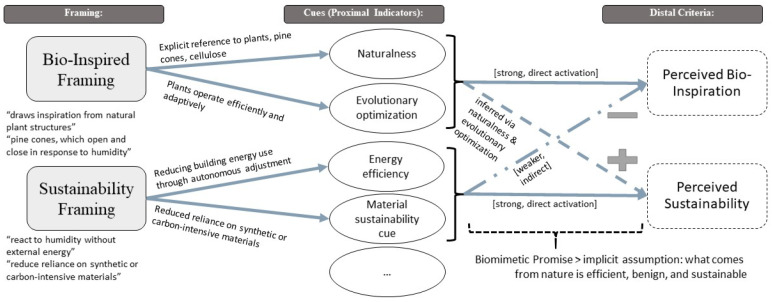
Conceptual representation of cue-based inference processes in the two framing conditions, grounded in Brunswik’s Lens Model. The Bio-Inspired and Sustainability Framings provide distinct sets of proximal cues—such as naturalness, evolutionary optimization, energy efficiency, and material sustainability—that differ in their perceived diagnosticity for judging distal criteria. These cues are differentially weighted when forming judgments about perceived bio-inspiration and perceived sustainability. The diagram also illustrates how the biomimetic promise—an implicit assumption that nature-based designs are efficient, benign, and sustainable—modulates cue utilization by strengthening indirect inferences from naturalness and evolutionary optimization cues to sustainability judgments.

The present study examined whether a biomimetic promise—understood here as the implicit expectation that biological inspiration should tend to imply sustainability—is mirrored in lay inferences in a vignette-based evaluation of a bio-inspired self-shading façade. While framing successfully shifts public perception toward the intended attribute, bio-inspiration and sustainability are perceived as cognitively distinct and potentially competing constructs. The framing manipulations successfully modulated the salience of the targeted attributes: Specifically, the bio-inspired framing significantly heightened the perceived bio-inspiration of the self-shading façade, while the sustainability framing selectively elevated its perceived ecological sustainability, that is, participants’ judgments of the façade’s environmental friendliness, resource-saving character, and comparative environmental benefit as captured by the PES items. These findings align with the core tenets of framing research, confirming that even brief, standardized textual stimuli can exert a powerful influence on public perception by prioritizing specific cognitive pathways and driving subsequent judgments [29]. Importantly, the observed divergence between the framing conditions supports a more complex interpretation of how laypersons evaluate bio-inspired technologies rather than a positive “halo effect”—where a favorable impression of one trait leads to biased positive assumptions about others [68]. Instead laypersons appear to reallocate evaluative weight toward the highlighted dimension at the explicit expense of the alternative. This phenomenon, known as “negative spillover”—where emphasizing one pro-environmental benefit inadvertently reduces support for another [69]. As shown in Figure 4 this is in line with a cue-utilization perspective (e.g., Brunswik’s Lens Model [31]), where framings can be interpreted as shifting which proximal cues participants treat as informative when inferring distal judgment like the perceived bio-inspiration or sustainability.

### Theoretical Explanation for Negative Spillover

The observed negative spillover effect directly contradicts the biomimetic promise, suggesting that in lay cognition, a nature-derived origin does not automatically function as a proxy for sustainability [27]. This divergence may be explained by contrast effects [70], where the accentuation of one salient attribute—such as biological inspiration—effectively suppresses the cognitive salience of alternative dimensions like sustainability. This suggests a process of cognitive partitioning, wherein laypersons categorize “biological inspiration” (focused on design process and origin) and “environmental benefit” (focused on functional outcome and utility) as distinct, independent evaluative criteria.

Further the biomimetic promise—the normative ideal that nature-derived solutions are inherently more sustainable—could be undermined by psychological distancing [71]. The biomimetic narrative, often framed as a high level socio-technical imaginary may feel psychologically distant to the public, particularly in domains like architecture [27].

## 5. Limitations and Considerations for Future Research

Despite the theoretical implications of these findings, several limitations delineate clear directions for future research. First, the exclusive use of text-based vignettes necessarily simplifies complex technological systems; future work could incorporate richer, multimodal stimuli (e.g., images, interactive 3D renderings, or short videos) to approximate more realistic encounters with emerging technologies while retaining experimental control [72,73]. Second, external validity is constrained by the use of a single engineered building technology presented as a pinecone-inspired self-shading façade. Accordingly, the present results should be interpreted as evidence about lay inferences in this specific architectural vignette context, rather than as field-level claims about biomimetics. Moreover, the stimulus necessarily contains nature-analogy content (e.g., pine cones, plant motion) that may act as an independent cue: such imagery can prime naturalness associations that influence perceived sustainability even when factual sustainability information is held constant. While the “natural-is-better” bias seems robust in bodily-applied domains like food or cosmetics [21], our results suggest that follow-up studies in context of bio-inspired technologies is needed: (i) vary the presence versus absence of explicit nature analogies, (ii) include multiple bio-inspired technologies with weaker or non-visual biological references beyond the architectural context. Further longitudinal designs are needed to test the durability of framing effects and whether initial impressions attenuate or consolidate as people receive additional technical information over time.

Critically, future research could adopt the lens-model perspective illustrated in Figure 4 to disentangle the cognitive mechanisms underlying these evaluations. According to Brunswik’s framework [31], individuals infer distal criteria (sustainability) from proximal cues (naturalness, evolutionary optimization). Our findings suggest a mismatch between “cue utilization” and “ecological validity” in the context of bio-inspired engineering. Future studies could systematically vary the diagnosticity and salience of these cues to determine the boundary conditions of the biomimetic promise—specifically testing whether nature-related cues are over- or under-utilized under conditions of high uncertainty or low technical knowledge [28].

Finally, incorporating moderators such as environmental values, technological optimism, and cross-cultural comparisons beyond UK samples will be essential to determine if the observed cognitive distinction between bio-inspiration and sustainability is a universal phenomenon or context-dependent.

## 6. Conclusions

Our findings demonstrate that while framing significantly shapes public perceptions of bio-inspiration and sustainability, there is no empirical support for thesustainability-oriented biomimetic promise as a default lay inference in the domain of this pinecone-inspired self-shading façade. Contrary to the assumption that nature-derived origins automatically signal ecological superiority, our results indicate that bio-inspiration and sustainability are perceived as cognitively distinct, and at times contradictory, constructs in this architectural scenario. The observed negative spillover—where emphasizing one attribute significantly undermines the other—challenges the generalizability of the nature-is-better bias typically found in bodily-applied consumer goods [21,27,32].

Accordingly, the present findings support the hypothesis that the default lay assumption associated with the sustainability-oriented biomimetic promise may not apply, and in this façade context may even reverse under framing. While these results do not justify unrestricted field-level claims about all biomimetic technologies, they do indicate that, for lay audiences evaluating this case, “bio-inspired” and “sustainable” are not automatically integrated into a single positive representation but can function as separable cues. In this sense, the study provide evidence against the assumption that nature-inspired design is inherently perceived as more sustainable [2,16]. Consequently, we argue that science communication should shift away from abstract biological metaphors and toward an “epistemic grammar” that prioritizes concrete functional outcomes and situated scientific practices [13]. By grounding the value of bio-inspired innovations in measurable engineering performance and local utility rather than biological essence, developers can mitigate unintended negative inferences and foster more transparent, evidence-driven public trust in emerging sustainable technologies.

## Figures and Tables

**Table 1:** Descriptive Statistics, Factor Loadings for the Perceived Ecological Sustainability Scale.

Item	Loading	Mean	SD	Skew.	Kurt.
The Self-Shading Facade is environmentally friendly.	0.880	5.57	1.20	−0.59	−0.27
The Self-Shading Facade has a positive impact on the environment in that it extends the life of discarded materials.	0.548	4.99	1.39	−0.51	−0.03
The Self-Shading Facade helps to save resources.	0.828	5.52	1.23	−0.64	−0.10
The Self-Shading Facade has more environmental benefits compared to similar products.	0.862	5.50	1.20	−0.53	−0.20

*Note.* Loadings are standardized CFA factor loadings. Skewness values reflect deviations from symmetry (0 = perfectly symmetric). Kurtosis was adjusted by subtracting 3, so that 0 indicates a normal distribution.

**Table 2:** Descriptive Statistics, Factor Loadings for the Perceived Bio-Inspiration Scale.

Item	Loading	Mean	SD	Skew.	Kurt.
Visual Resemblance to Nature (VRtN):					
The Self-Shading Facade sounds like something I might find in the natural world.	0.81	3.44	1.04	−0.56	−0.35
The Self-Shading Facade’s description reminds me of an animal, plant, or natural environment.	0.87	3.48	1.10	−0.64	−0.40
In this Self-Shading Facade I can easily imagine forms that imitate living creatures or natural patterns.	0.78	3.45	0.98	−0.47	−0.36
Imagining the look of the Self-Shading Facade, I do not think of examples from the natural world. (reverse-coded)	0.66	3.26	1.10	−0.27	−0.85
Intentionality & Perceived Inspiration (IPI):					
It seems clear the designers deliberately took ideas from living nature for the Self-Shading Facade.	0.89	3.78	0.97	−0.72	0.10
The Self-Shading Facade does not seem to be directly modeled on observations of living beings. (reverse-coded)	0.45	3.40	1.06	−0.32	−0.73
I feel the designers of the Self-Shading Facade made a purposeful attempt to take inspiration from the natural world.	0.85	3.93	0.94	−0.84	0.35
I believe the Self-Shading Facade was planned with examples from living nature firmly in mind.	0.89	3.79	0.99	−0.72	0.02
Perceived Naturalness (PN):					
The Self-Shading Facade gives off a natural vibe, like it belongs in a natural environment.	0.87	3.54	0.97	−0.60	−0.10
The Self-Shading Facade fits seamlessly with natural surroundings when I imagine it in place.	0.66	3.55	0.92	−0.38	−0.31
Overall, the Self-Shading Facade comes across as a naturally derived, rather than purely engineered, object.	0.72	3.15	1.06	−0.23	−0.87

*Note.* Reverse-coded items are marked accordingly. Loadings are standardized CFA factor loadings. Skewness values reflect deviations from symmetry (0 = perfectly symmetric). Kurtosis was adjusted by subtracting 3, so that 0 indicates a normal distribution.

**Table 3:** Means and Standard Deviations of PBS (Overall and Subscales) and PES Ratings by Framing Condition.

Framing Condition	PBS	PN	IPI	VRtN	PES
Bio-inspired	3.92 (0.51)	3.55 (0.74)	4.29 (0.55)	3.82 (0.68)	4.83 (1.06)
Neutral	3.48 (0.70)	3.42 (0.81)	3.59 (0.75)	3.41 (0.82)	5.46 (0.95)
Sustainable	3.18 (0.82)	3.27 (0.90)	3.31 (0.80)	2.99 (0.94)	5.88 (0.84)

*Note.* Values represent means, with standard deviations presented in parentheses. PBS = Perceived Bio-Inspiration Scale (overall score); PN = Perceived Naturalness subscale; IPI = Intentionality and Perceived Inspiration subscale; VRtN = Visual Resemblance to Nature subscale; PES = Perceived Ecological Sustainability Scale.

**Table 4:** Regression Results for the Framing × Rating-Type Interaction Model.

Predictor	Estimate (SE)
Main Effects	
Bio-inspired framing vs. neutral ($\beta_{1}$)	0.587 *** (0.093)
Sustainable framing vs. neutral ($\beta_{2}$)	−0.388 *** (0.092)
Rating type: PES vs. PBS ($\beta_{3}$)	0.131 (0.092)
Interaction Terms	
Bio-inspired framing × PES ($\beta_{4}$)	−1.197 *** (0.131)
Sustainable framing × PES ($\beta_{5}$)	0.789 *** (0.130)
Intercept	
Constant ($\beta_{0}$)	−0.064 (0.065)
Observations	1176
$R^{2}$	0.166
Adjusted $R^{2}$	0.163
Residual SD	0.915 (df = 1170)
F-statistic	46.68 *** (df = 5, 1170)

*Note.* Reference levels: neutral framing (between-subjects) and PBS ratings (within-subjects). *** p<0.01.

## Data Availability

Materials, data, and code for data analyses, can be downloaded at: https://github.com/FennStatistics/Article_BiomimeticPromise (accessed on 12 March 2026).

## Abbreviations

The following abbreviations are used in this manuscript:

PBS	Perceived Bio-Inspiration Scale
PES	Perceived Ecological Sustainability Scale
SD	Standard Deviation
EMM	Estimated Marginal Mean
CFA	Confirmatory Factor Analysis
EFA	Exploratory Factor Analysis
MLR	Robust Maximum Likelihood Estimator
CFI	Comparative Fit Index
TLI	Tucker–Lewis Index
RMSEA	Root Mean Square Error of Approximation
SRMR	Standardized Root Mean Square Residual
AIC	Akaike Information Criterion
BIC	Bayesian Information Criterion
df	Degrees of Freedom

## Appendix A. Vignettes of Self-Shading Façade

Neutral Framing. The Self-Shading Facade is a recent development in innovative materials for architecture. Its surface is made up of hundreds of small, curved modules suspended across window frames. These modules are made of layered materials that bend in response to changing humidity. As humidity rises or falls, the facade’s individual elements autonomously curl or flatten, adjusting how much light and heat pass through. The movement is driven by the structure of the materials themselves, without the need for motors or electronics. This technology contributes to innovative architecture materials and building energy regulation. It is a functional system designed using recent advances in materials science.

Bio-inspired Framing. The Self-Shading Facade is a recent development in innovative materials for architecture. Its surface is made up of hundreds of small, curved modules suspended across window frames. These modules are made of layered materials that bend in response to changing humidity. The design draws inspiration from natural plant structures, particularly pine cones, which open and close in response to humidity. The layers are inspired by how cellulose fibers are arranged in these plants to guide the direction of bending. This technology brings ideas from the natural world into architectural innovation. Its function and movement are grounded in bio-inspired design.

Sustainable Framing. The Self-Shading Facade is a recent development in innovative materials for architecture. Its surface is made up of hundreds of small, curved modules suspended across window frames. These modules are made of layered materials that react to humidity changes without needing external energy. Their composition helps reduce reliance on synthetic or carbon-intensive materials. Because the facade adjusts shading based on weather, it offers a way to reduce energy use in buildings. This technology supports climate-friendly architectural innovation and resource efficiency. Its passive, energy-autonomous operation reflects sustainable design principles.
